# Computational prediction of RNA modification site-disease associations: a systematic review

**DOI:** 10.1093/bib/bbag388

**Published:** 2026-07-17

**Authors:** Chunyan Ao, Shihu Jiao, Xi Su, Ying Ju, Quan Zou, Linpei Jia

**Affiliations:** Institute of Fundamental and Frontier Sciences, University of Electronic Science and Technology of China, No. 2006 Xiyuan Avenue, West Hi-Tech Zone, Chengdu 611731, Sichuan, China; Yangtze Delta Region Institute (Quzhou), University of Electronic Science and Technology of China, No. 1 Chengdian Road, Kecheng District, Quzhou 324003, Zhejiang, China; Institute of Fundamental and Frontier Sciences, University of Electronic Science and Technology of China, No. 2006 Xiyuan Avenue, West Hi-Tech Zone, Chengdu 611731, Sichuan, China; Yangtze Delta Region Institute (Quzhou), University of Electronic Science and Technology of China, No. 1 Chengdian Road, Kecheng District, Quzhou 324003, Zhejiang, China; The Affiliated Foshan Women and Children Hospital, Guangdong Medical University, Foshan 528000, China; School of Informatics, Xiamen University, No. 422 South Siming Road, Siming District, Xiamen 361005, Fujian, China; Institute of Fundamental and Frontier Sciences, University of Electronic Science and Technology of China, No. 2006 Xiyuan Avenue, West Hi-Tech Zone, Chengdu 611731, Sichuan, China; Yangtze Delta Region Institute (Quzhou), University of Electronic Science and Technology of China, No. 1 Chengdian Road, Kecheng District, Quzhou 324003, Zhejiang, China; Department of Nephrology, Xuanwu Hospital, Capital Medical University, No. 45 Changchun Street, Xicheng District, Beijing 100053, Beijing, China

**Keywords:** RNA modification sites, diseases, machine learning, association prediction

## Abstract

With the development of epitranscriptomics, studies have shown that RNA modification sites are closely related to many diseases. Because experimental validation is time-consuming and labor-intensive, an increasing number of studies have developed computational methods to predict potential associations between RNA modification sites and diseases. In this review, we summarize recent progress in predicting RNA modification site-disease associations, with a focus on common modifications such as m6A, m1A, and m7G. First, we systematically summarize commonly used databases and data sources and outline approaches for constructing similarity information for modification sites and diseases. We then review existing prediction methods—including network-based strategies, matrix completion, and machine learning—and discuss their typical advantages and limitations. Finally, we highlight key challenges in this field, including limited known associations, data imbalance, unclear definitions of negative samples, inconsistent evaluation standards across studies, and limited interpretability and experimental validation. We also suggest future directions, such as expanding high-quality datasets, integrating multi-omics data, establishing unified evaluation pipelines, and strengthening experimental validation. We hope this review provides a clear overview and practical guidance for studies on the associations between modification sites and diseases and supports the development of more reliable prediction methods.

## Introduction

RNA post-transcriptional modifications are an essential component of the epitranscriptome and have received increasing attention in biomedical research in recent years [1]. With continual advances in detection and profiling methods, researchers have identified more than 170 RNA modification types across diverse RNA species, including mRNA, lncRNA, tRNA, and rRNA [2, 3]. These methodological advances have substantially broadened epitranscriptomic research and accelerated interest in RNA modification-mediated regulation. Meanwhile, the widespread application of high-throughput sequencing in transcriptomics has facilitated the establishment and refinement of several databases related to RNA modifications, such as RMBase v2.0 [4], RMDisease v2.0 [5], m7GHub [6], MODOMICS [3], and RMVar v2.0 [7]. These resources support ongoing data accumulation and further enhance the understanding of the regulatory mechanisms and biological functions associated with RNA modifications. Currently, the most extensively studied types include N6-methyladenosine (m6A), N7-methylguanosine (m7G), N1-methyladenosine (m1A), and 5-methylcytidine (m5C), which are broadly conserved across archaea, bacteria, fungi, plants, animals, and humans [3, 8]. Many RNA modifications are regulated by specific protein factors—often termed ‘writers’, ‘readers’, and ‘erasers’—that influence key RNA processes such as splicing, stability, nuclear export, translation efficiency, and degradation [9–11]. Through these mechanisms, RNA modifications contribute to diverse biological programs and disease pathogenesis, including tumorigenesis, immune regulation, neurological dysfunction, and metabolic disorders [12–15].

**Figure 1:**
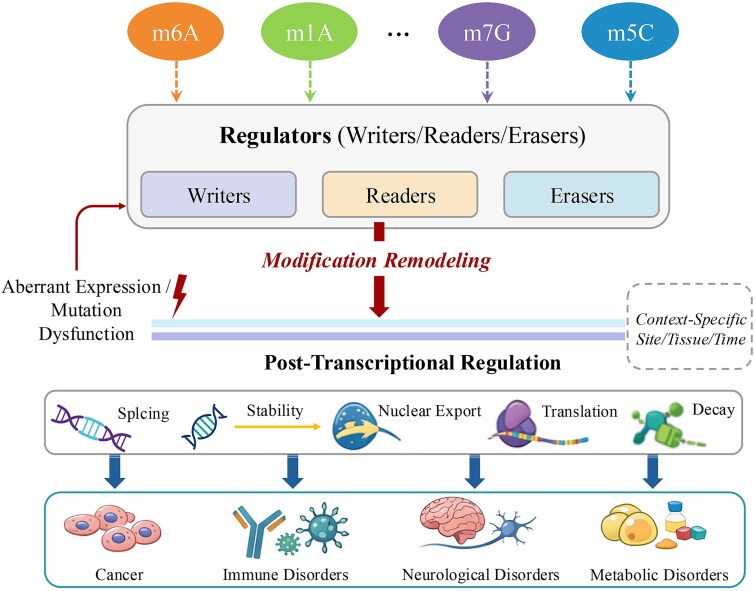
Conceptual overview of the relationship between RNA modification sites and diseases.

Increasing evidence suggests that changes in RNA modification levels and disruptions in their regulatory networks are closely associated with the development and progression of various diseases (Fig. 1) [16–18]. Under normal physiological conditions, RNA modifications constitute dynamic and reversible layers of post-transcriptional control that help maintain appropriate gene expression programs [17]. In pathological contexts, aberrant expression, mutation, or functional impairment of writers, readers, and erasers can drive global shifts in modification abundance or site-specific redistribution across transcripts, thereby reshaping downstream gene-regulatory programs and cellular states [16, 19]. These changes can affect RNA splicing, stability, nuclear export, and translation efficiency. Such disruptions may further interfere with key biological processes such as cell cycle control, DNA repair, immune responses, neural function, and metabolic balance, and have been linked to cancers, immune disorders, neurodegenerative diseases, and metabolic syndromes. For example, m6A is the most common and well-studied internal RNA modification. It plays critical roles in various diseases by regulating transcript stability, cell fate decisions, and translation [20, 21]. Internal m7G modifications in mRNA, as well as dysregulation of its regulators, such as METTL1 and WDR4, have been associated with multiple cancers, and pathogenic WDR4 variants can also cause neurodevelopmental disorders [12, 22–25]. m1A, though less abundant, is enriched in the 5′ UTR, tRNA, and mitochondrial mRNAs and has been linked to cardiovascular disease, cancer, and mitochondrial metabolic disorders [13, 26, 27]. Therefore, investigating the associations between RNA modification sites and diseases is essential for understanding post-transcriptional regulation in disease mechanisms and for identifying key regulatory events. Modification sites with unknown functions may hold clues to disease-associated regulatory pathways and could serve as potential therapeutic targets or molecular biomarkers, offering new avenues for diagnosis and treatment.

However, the associations between RNA modification sites and diseases are often site-specific, tissue-dependent, and dynamically regulated in time and space [17, 28]. Relying solely on traditional wet-lab experiments for one-by-one validation is not only costly, time-consuming, and inefficient, but also prone to sample heterogeneity, which can lead to inconsistent results [29, 30]. Therefore, developing efficient computational methods to systematically predict potential associations between RNA modification sites and diseases has become a key research focus. Such approaches not only facilitate the identification of critical modification sites and regulatory pathways but also improve our understanding of the epitranscriptomic mechanisms underlying disease. In recent years, the rapid development of machine learning technologies has significantly broadened their applications in RNA modification studies-especially in predicting associations between RNA modification sites and diseases, where they have shown promising performance [31–34]. To date, a variety of computational methods have been proposed for RNA modification site-disease association prediction, which can be broadly categorized into graph propagation/network inference approaches, traditional machine learning models, and deep learning frameworks [35–37]. These methods typically integrate heterogeneous data sources, including RNA modification annotations, regulatory protein information, gene expression profiles, disease ontologies, and molecular interaction networks, to model potential functional relationships and regulatory mechanisms. Therefore, a systematic review of recent advances in predicting associations between RNA modification sites and diseases is needed to help researchers better understand the design and application of current methods and to guide future research in this field.

In this review, we systematically summarize recent advances in predicting associations between RNA modification sites and diseases, with a focus on major computational methods and their modeling frameworks proposed in recent years. Based on model design strategies and core algorithm frameworks, we categorize existing approaches into three methodological directions: machine learning-based methods, matrix completion-based methods, and network propagation-based methods. For each category, we introduce the key ideas, input feature construction strategies, and typical application scenarios, and we summarize the advantages and limitations commonly reported in the literature, such as limited known associations, data sparsity, feature construction bias, and interpretability challenges. In addition, we review commonly used data resources and further discuss current challenges in the field, such as class imbalance and limited cross-platform generalization. Finally, we outline future research directions, including multi-modal feature integration, unified modeling of different RNA modification types, weakly supervised learning, and improved model interpretability, aiming to provide a comprehensive reference for subsequent studies.

## Data resources and evaluation

For predicting associations between RNA modification sites and diseases, high-quality data and informative similarity features are essential for developing computational methods. In this review, we briefly summarize commonly used databases and describe how to construct multi-level similarity networks among RNA modification sites, diseases, and genes, thereby providing basic data and feature support for subsequent prediction models. Furthermore, this section provides an overview of commonly used model evaluation strategies, including validation settings, negative sample construction, and performance evaluation metrics.

### RNA modification and variation databases

High-quality datasets are an important foundation for studies on predicting RNA modification site-disease associations. Therefore, we systematically reviewed and summarized existing data on RNA modification sites and their related functional variants, and the information on relevant databases is listed in Table 1. Next, we briefly introduce these databases to provide data references and support for related research. Existing databases on RNA modification sites and related variants mainly fall into two categories. The first category collects and organizes transcriptome-wide RNA modification site information. The second category focuses on genetic variants related to RNA modifications and their associations with diseases or traits.

**Table 1:** RNA modification and variation databases.

**Databases**	**Database type**	**Modification types**	**Main contents**	**URL**
RMBase v2.0 [4]	RNA modification site database	Multiple RNA modification types, including m6A, m1A, m5C, Ψ, and others	Collects transcriptome-wide RNA modification site coordinates across multiple species, with basic annotations such as modification type, motifs, and associated RBPs/SNPs.	https://rna.sysu.edu.cn/rmbase/
m7GHub v2.0 [38]	Integrated m7G platform	m7G	Updated, comprehensive resource for m7G epitranscriptome data, including m7GDB (~430 k sites in 23 species) and its disease/variant module m7GDiseaseDB, with web tools for m7G prediction and variant-effect analysis.	https://www.rnamd.org/m7GHub2/
RMVar v2.0 [7]	RNA modification variant database	Nine major RNA modification types, including m6A, m1A, m5C, m7G, Ψ, and others	Updated database of genetic variants associated with nine types of RNA modifications in human and mouse (~2.08 million RM-associated variants at four evidence levels), with detailed annotations of genomic context, conservation, molecular functions (RBP binding, RNA–RNA interactions, splicing, circRNA) and disease links from GWAS and ClinVar.	https://rmvar.renlab.cn/
RMDisease v2.0 [5]	Variant–RNA modification–disease/trait database	Multiple RNA modification types associated with human diseases and traits	Records variants that affect different RNA modifications and their associations with human diseases and complex traits.	https://www.rnamd.org/rmdisease2/
DirectRMDB [2]	Nanopore DRS-based RNA modification database	Multiple RNA modification types detected or inferred from Nanopore direct RNA sequencing data	Stores RNA modification sites detected by Nanopore direct RNA sequencing and provides modification abundance and confidence at transcript/isoform level across species and modification types.	http://www.rnamd.org/directRMDB/
MODOMICS [3]	RNA modification knowledgebase	Comprehensive coverage of more than 170 RNA modification types	Summarizes chemical structures, names, positions in different RNAs, and related enzymes and metabolic pathways for many types of RNA modifications.	https://genesilico.pl/modomics/
RADAR [39]	A-to-I RNA editing database	A-to-I RNA editing	Collects A-to-I RNA editing sites in human, mouse and fly, and provides detailed site annotations and tissue/sample-specific editing levels.	http://RNAedit.com ^a^

^a^Website is not available at the time of writing.

In terms of resources for RNA modification site information, RMBase v2.0 [4] is a comprehensive database that collects transcriptome-wide RNA modification sites across multiple species and provides basic annotations such as modification types and sequence motifs. It also integrates information on related RBPs and SNPs, so it is often used to build site-gene mappings and site similarity features for downstream functional analysis. m7GHub v2.0 [38] is a dedicated platform for m7G modification sites and contains about 430 000 m7G sites from 23 species. It also offers disease- and variant-related modules such as m7GDiseaseDB to link site information to phenotypes. In addition, it provides online tools for m7G prediction and variant effect analysis to support data use and result reproducibility. DirectRMDB [2] is built from sequencing-based detection results and reports not only a list of modification sites but also modification abundance and confidence at the transcript and isoform levels. Because it is based on direct RNA sequencing data, it is useful for assessing the consistency and reliability of sites identified by different analysis pipelines. MODOMICS [3] is a structured database for RNA modifications that summarizes the chemical structures and standardized names of modified ribonucleosides and provides typical positions and annotations in different RNA sequences. It also includes information on related enzymes, reaction steps, and biosynthetic pathways to support mechanistic interpretation. RADAR [39] mainly focuses on A-to-I RNA editing and collects editing sites from humans, mouse, and fruit flies. It provides manually curated site annotations such as the associated gene, genomic region, repeat elements, and conservation. It also integrates tissue- or sample-specific editing levels from published RNA-seq data to enable comparisons across tissues or conditions.

For resources on modification-related genetic variants, RMVar v2.0 [7] systematically integrates functional variants linked to nine major types of RNA modifications in humans and mouse. It organizes these variants into four evidence levels. It annotates variants using MeRIP-seq analysis, allele-specific variant detection, and deep learning-based prediction. It also provides functional impact information and integrates disease-related evidence. RMDisease v2.0 [5] focuses on evidence linking genetic variants, RNA modifications, and diseases or traits. It collects variants that may cause a gain or loss of modification sites and links them to corresponding disease and trait information. It provides phenotype-oriented search options, including searches by gene, genomic region, rs ID, disease phenotype, and trait. It also allows exporting association records for candidate variant screening, mechanistic analysis, and result validation.

### Similarity features and network construction

For the diverse input information used in existing prediction models, we first classified the biological and network features commonly used for predicting RNA modification site-disease associations. These features mainly fall into three categories: RNA modification site-related features, disease-related features, and association- or network-level features. The following sections further describe how these features are transformed into site similarity, disease similarity, multi-source similarity, and heterogeneous network structures and how they are used for downstream association prediction.

#### Similarity features

(1) Site similarity

Site-site similarity is commonly derived from the local sequence context around each modification site and its derived representations. Typical inputs include k-mer statistics, physicochemical descriptors, and embedding vectors, which form a feature vector ${\mathbf{x}}_i$ for site ${s}_i$. Pairwise similarity between ${\mathbf{x}}_i$ and ${\mathbf{x}}_j$ is then used to build a site similarity matrix ${W}^{SS}\in{\mathbb{R}}^{m\times m}$. Frequently used measures include cosine similarity, Jaccard similarity for discrete feature sets, and Pearson correlation.

Beyond sequence, functional-context similarity is also widely used. It relies on regulatory annotations such as the 5′ UTR, 3′ UTR, and CDS regions, and proximity to splice-related regions, RNA-binding protein binding regions, and miRNA target sites. These signals can be converted into site features or used to weight edges, providing an indirect but informative description of site context. Public resources such as RMVar v2.0 provide annotations related to RBP interactions, RNA–RNA interactions, splicing events, and circRNAs. It also integrates disease evidence from ClinVar and GWAS, enabling the construction of context-aware similarity features [7].

When direct site-disease links are limited, variant perturbation signals can provide complementary evidence. Disease-related variants that may cause gain or loss of a modification site are often mapped to sites and then used to connect sites that share similar perturbation patterns, such as similar variant types or shared LD or tagSNP evidence. Databases such as m7GHub additionally organize sites and variants by evidence level, which can be used to weight edges [38].

(2) Disease similarity

Disease similarity is usually computed from multiple sources, including phenotype or symptom profiles, ontology-based semantics, and mechanism-level information such as genes, pathways, and drugs. Ontology-based similarity commonly relies on information content (IC):


(1) \begin{equation*} IC(t)=-\log p(t) \end{equation*}

Classic measures such as Resnik and Lin are defined based on shared ancestors in the ontology DAG [40, 41]. In practice, many studies compute these scores using existing packages such as DOSE and then combine them with phenotype and molecular similarity to improve coverage [42]. Disease Ontology is widely used as a controlled vocabulary that supports semantic similarity computation at the term level [43].

(3) Fusion of multi-source similarity

Similarity is often constructed from multiple views for both sites and diseases. A common approach is to linearly fuse view-specific similarity matrices:


(2) \begin{equation*} {\tilde{W}}^{SS}=\sum_{v=1}^{V_S}{\alpha}_v{W}_{(v)}^{SS},{\tilde{W}}^{DD}=\sum_{u=1}^{V_D}{\beta}_u{W}_{(u)}^{DD} \end{equation*}

The weights are non-negative and sum to 1. Alternatively, view-specific networks can be kept as separate relations and integrated during graph modeling. This preserves view-specific signals but increases relation complexity. A similarity network fusion strategy can be used to integrate multiple similarity networks [44].

Because similarity scores may have different scales, normalization is typically applied, such as symmetric normalization. Since fused similarity matrices are often dense, sparsification and denoising are almost always used to suppress weak edges. Thresholding and k-nearest-neighbor (KNN) edge retention are common choices. KNN graphs are frequently symmetrized using a union rule to reduce directional instability. The parameters, including the threshold $\tau$ and the neighbor number $k$, are usually chosen to balance noise suppression and information retention, often guided by validation or sensitivity analysis. Graph Laplacian formulations and kNN graph construction are standard components in manifold and graph-based learning [45].

#### Network construction

(1) Association network

Known site-disease links are represented by an association matrix $A\in{\left\{0,1\right\}}^{m\times n}$ or, equivalently, a bipartite graph ${G}_{SD}=\left(S,D,E\right)$, where $m=\mid S\mid$ and $n=\mid D\mid$ denote the numbers of modification sites and diseases, respectively. Let ${A}_{ij}=1$ if a reported association exists between site ${s}_i$ and disease ${d}_j$, and ${A}_{ij}=0$ otherwise. In most datasets, entries with ${A}_{ij}=0$ are unlabeled rather than confirmed negatives, so the task is often formulated under a positive-unlabeled (PU) or weakly supervised setting [46]. Evidence supporting the edges can be heterogeneous: a small fraction comes from direct site-level experiments, whereas many links are inferred indirectly via variant-based mappings that connect site gain/loss events to disease or trait evidence. To reduce noise, edges are often stratified or weighted by evidence strength.

(2) Heterogeneous network

A standard practice is to integrate the within-type similarity networks and the site-disease association network into a unified heterogeneous graph. Let ${W}^{SS}\in{\mathbb{R}}^{m\times m}$ and ${W}^{DD}\in{\mathbb{R}}^{n\times n}$ denote the sparsified site-site and disease-disease similarity matrices, respectively, and let $A\in{\left\{0,1\right\}}^{m\times n}$ denote the site-disease association matrix. A common block adjacency form is:


(3) \begin{equation*} H=\left[\begin{array}{@{}cc@{}}{\mathrm{W}}^{SS}& A\\{}{A}^{\top }& {\mathrm{W}}^{DD}\end{array}\right]\in{\mathbb{R}}^{\left(m+n\left)\times \right(m+n\right)} \end{equation*}

This representation jointly encodes site-site similarity, disease-disease similarity, and known site-disease links, and enables inference on a unified graph, such as propagation, completion, or ranking.

(3) Multi-layer node network

When $A$ is extremely sparse, many studies further extend the bipartite network into a multi-layer heterogeneous network by inserting intermediate layers such as variants or genes. Indirect paths $S\to V/G\to D$ can connect site perturbations to disease evidence and provide a clearer evidence chain.

Let $V=\left\{{v}_1,\dots, {v}_k\right\}$ and $G=\left\{{g}_1,\dots, {g}_p\right\}$. Cross-layer relations can be described by matrices ${A}^{SV},$  ${A}^{VD}$, ${A}^{SG}$, and ${A}^{GD}$. A variant–gene matrix ${A}^{VG}$ can be included when variant–gene links are available. These matrices can be either binary or edge-weighted to reflect confidence. A unified supra-adjacency matrix is often used to represent all layers in a single block matrix. Intermediate layers typically act as bridges. Within-type similarity blocks for variants or genes are added only when reliable similarity information exists. This design helps retain connectivity for downstream inference even when direct associations are highly sparse and improves interpretability through indirect evidence chains. Moreover, because confirmed negative associations are rarely available, some studies adopt PU learning to train classifiers using known positives and unlabeled pairs [47].

### Model evaluation

Model performance evaluation is a crucial step in studies of RNA modification site-disease association prediction. Due to the limited number of experimentally verified site-disease associations and the difficulty in defining confirmed negative samples, the reported performance of different studies can be affected by data splitting strategies, negative sample construction, and the choice of evaluation metrics. Therefore, this section briefly outlines commonly used evaluation strategies.

(1) **Validation settings and data splitting strategies:** Most existing prediction methods use k-fold cross-validation or leave-one-out cross-validation to evaluate model performance. Random pairwise splitting is simple, but it may cause the same disease or modification site to appear in both the training and test sets, thereby affecting the evaluation of model generalization ability. Therefore, some studies also adopt disease-level splitting strategies, such as *de novo* disease tests or leave-one-disease-out validation, to evaluate the predictive ability of models when known association information for a given disease is unavailable.
(2) **Negative sample construction and unlabeled sample handling:** Negative sample construction is also important for model training and evaluation. Known site-disease associations are usually regarded as positive samples, whereas unobserved site-disease pairs are not equivalent to true negative samples. Random negative sampling is simple, but it may introduce false negatives, and the results can be affected by the sampling ratio, sampling strategy, and random seed. Therefore, some studies adopt strategies such as PU learning, reliable negative sample selection, or weighted learning to reduce the bias caused by the uncertainty of negative samples.
(3) **Evaluation metrics:** Common evaluation metrics include the area under the receiver operating characteristic curve (AUC), the area under the precision–recall curve (AUPR), accuracy, precision, recall, and F1-score. AUC can reflect the overall discriminative ability of a model, but in RNA modification site-disease association prediction tasks with class imbalance, it may not sufficiently reflect the model’s ability to identify positive samples. Therefore, AUPR and top-ranked candidate analysis are also often used to evaluate model performance, especially for candidate site ranking and prioritization tasks.

## Methods for predicting RNA modification site-disease associations

**Figure 2:**
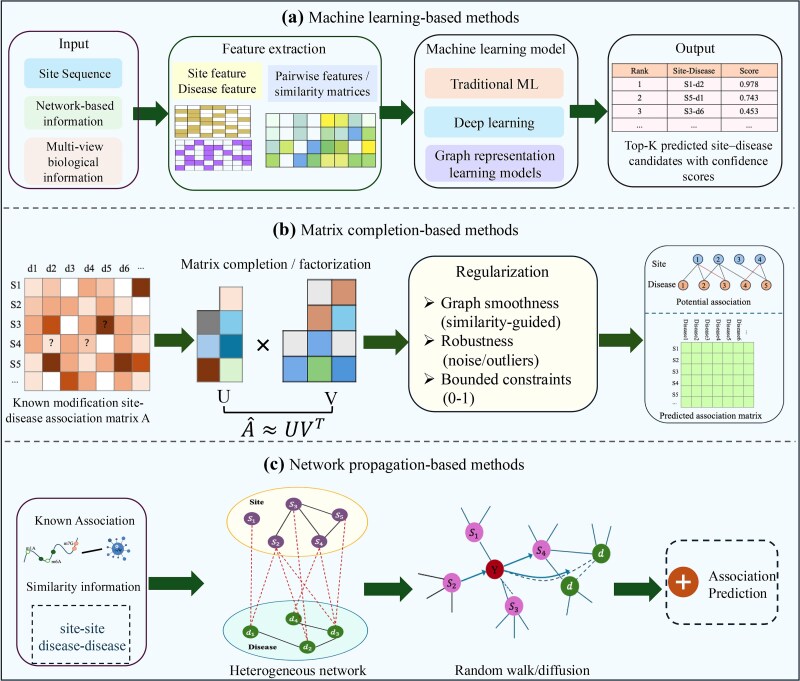
Overview of computational frameworks for predicting RNA modification site-disease associations: (a) machine learning-based methods, (b) matrix completion-based methods, and (c) network propagation-based methods.

In this review, we summarize computational methods for predicting RNA modification site-disease associations, as shown in Table 2. Based on model design strategies and core algorithm frameworks, existing methods can be grouped into three categories: (i) machine learning-based methods, (ii) matrix completion-based methods, and (iii) network propagation-based methods. The following sections introduce these three categories in order. The overall framework is shown in Fig. 2.

**Table 2:** Summary of RNA modification site-disease association prediction methods.

**Method**	**Modification type(s)**	**Data sources**	**Model framework**	**Evaluation strategy**	**Code / web server**	**Year**
DRUM [53]	m6A sites	Gene expression data, RNA methylation data, disease–gene associations, gene–m6A site associations, and disease similarity data	Multi-layer heterogeneous network integrating diseases, genes, and m6A sites with RWR	10-fold cross-validation, comparison with hypergeometric test-based method and random predictor, and literature-supported cancer-related site analysis	www.xjtlu.edu.cn/biologicalsciences/drum	2019
m7GDisAI [50]	m7G sites	m7GHub/m7GDiseaseDB-derived m7G–disease associations, genomic information of m7G sites, disease phenotype information, m7G site similarity, and disease semantic similarity	Heterogeneous m7G–disease network with matrix factorization	10 runs of 10-fold cross-validation, global/local LOOCV, and ovarian cancer case study	http://180.208.58.66/m7GDisAI/	2021
HN-CNN [48]	m7G sites	m7GDiseaseDB-derived m7G–disease associations, m7G site chemical similarity, and disease similarity	Heterogeneous network feature construction, CNN feature extraction, and XGBoost classifier	10 runs of 10-fold cross-validation with randomly sampled unknown pairs treated as negative samples; case study and GO analysis	Not available	2021
BRPCA [51]	m7G sites	m7G–disease association matrix, m7G chemical similarity, m7G CNF similarity, and disease similarity	Bounded robust principal component analysis incorporating similarity information	10-fold cross-validation, *de novo* disease test, ROC/PR analysis, and benchmark comparison	Not available	2021
SpBLRSR [52]	m7G sites	m7GDisAI-derived m7G–disease association matrix, m7G chemical similarity, m7G CNF similarity, and disease similarity	Schatten p-norm constrained bounded low-rank subspace recovery using an m7G–disease block matrix	10-fold CV, leave-one-disease-out cross-validation, ROC/PR analysis, benchmark comparison, noise contamination experiment, and breast cancer case study	https://github.com/jianiM/SpBLRSR	2022
m7GDP-RW [36]	m7G sites	m7GHub-derived gold standard m7G–disease associations, m7G site features, disease semantic information, and known association information	Similarity optimization and two-pass RWR on an m7G–disease heterogeneous network	10-fold cross-validation, ROC/PR analysis, comparison with existing methods, candidate ranking, and case studies	https://github.com/hyr0771/m7GDP-RW	2023
RMDGCN [35]	m1A sites	Collected m1A–disease associations, m1A site features, disease semantic similarity, and disease symptom similarity	Positive–unlabeled learning, heterogeneous network construction, and attention-based GCN	5-fold cross-validation using AUC and AUPR; breast cancer case study	https://github.com/lianliu09/RMDGCN	2023
PathoRM [49]	m6A and m7G sites	m6ADA and m7GDA datasets; RNA methylation host sequences and pathogenic disease descriptions	Large language model-based feature extraction, multi-view learning, graph neural network, adversarial training, and guilty-by-association-derived negative sampling	10-fold cross-validation on balanced and imbalanced datasets, multi-metric evaluation, Wilcoxon test, robustness comparison, and interpretability analysis	https://github.com/jianiM/PathoRM	2025

### Machine learning-based methods

Machine learning-based methods model the associations between RNA modification sites and diseases as either a pairwise scoring task or a binary classification task. The input is a candidate pair $\left({s}_i^m,{d}_j\right)$. The output is an association probability or a ranking score. Most methods build a joint representation for each candidate pair by integrating three sources of information: (i) site-related information, such as local sequence windows, sequence encodings/embeddings, and functional region annotations; (ii) disease-related information, such as semantic/ontology representations, phenotype or symptom similarity, and disease-related molecular information; and (iii) network-related information, such as site similarity ${W}^{SS}$, disease similarity ${W}^{DD}$, and the known association matrix $A$. Some methods further learn node embeddings from the heterogeneous graph.

A common design is to learn a function ${f}_{\theta }$ that maps the fused feature vector ${z}_{ij}$ to an association probability:


(4) \begin{equation*} {\hat{p}}_{ij}=\sigma \left({f}_{\theta}\left({z}_{ij}\right)\right),{z}_{ij}=\left[{x}_i\parallel{y}_j\parallel{x}_i\odot{y}_j\right] \end{equation*}

Here, ${x}_i$ and ${y}_j$ are feature vectors for the site and the disease. The Hadamard product term $\left({x}_i\odot{y}_j\right)$ models simple feature interactions. Other fusion operators are also used, such as bilinear pooling or neural attention.

A major difficulty is label ambiguity. Known pairs $\left({A}_{ij}=1\right)$ are positives, whereas pairs with ${A}_{ij}=0$ are unlabeled and may contain hidden positives. Many datasets are also highly imbalanced. Therefore, many studies use PU learning or weak supervision. A widely used practical strategy is to treat unlabeled pairs as negatives but reduce their influence. This can be done by loss weighting or adaptive sampling. A typical weighted objective is:


(5) \begin{equation*} \mathcal{L}=-\sum_{\left(i,j\right)\in \mathcal{P}}\log{\hat{p}}_{ij}-\lambda \sum_{\left(i,j\right)\in \mathcal{U}}\log \left(1-{\hat{p}}_{ij}\right) \end{equation*}

where $P=\left\{\left(i,j\right)\mid{A}_{ij}=1\right\}$ and $U=\left\{\left(i,j\right)\mid{A}_{ij}=0\right\}$ denote the positive and unlabeled sets, respectively, and $\lambda \ge 0$ controls the weight of the unlabeled term, where smaller $\lambda$ reduces the impact of potential false negatives in $U$. Although this strategy is simple and practical, it still mixes true negatives and hidden positives in $U$. Therefore, model calibration and external validation remain important.

Representative machine learning-based methods include HN-CNN, RMDGCN, and PathoRM. For example, to predict associations between m7G modification sites and diseases, Zhang *et al.* proposed HN-CNN [48]. HN-CNN uses m7GDiseaseDB as its data source. It integrates site similarity, disease similarity, and known site-disease associations into a heterogeneous network. For each candidate pair, it constructs pairwise features from the association matrix and the similarity matrices. Site similarity is computed from chemical-property features using Jaccard similarity. Disease similarity is obtained from DisSetSim. A CNN is used to extract higher-level representations from the sparse pair features, and XGBoost outputs association scores for prediction and ranking. Performance was evaluated using 10-fold cross-validation. In each fold, an equal number of unknown pairs were randomly sampled and treated as negative samples. The model achieved an average AUC of about 0.827. Overall, HN-CNN combines network structure information with deep feature learning and uses ensemble learning to improve stability. However, random sampling from unknown pairs may introduce label noise. The 1:1 positive-to-negative setting differs from the highly imbalanced real-world scenario. The method also relies on the quality of the similarity measures and the coverage of external knowledge. Biological evidence is still needed to interpret the predictions.

RMDGCN [35] is a computational method developed for m1A site-disease association prediction. It is formulated as link prediction on a heterogeneous network. For m1A site similarity, flanking sequences are tokenized into 3-mers and used to train a word2vec model, yielding a 100-dimensional vector for each site. Cosine similarity is then used to build the site similarity network, denoted as RRS in the original RMDGCN study. Disease similarity combines DOSE/DOID-based semantic similarity and symptom similarity into an integrated disease similarity matrix, referred to as DDS in the original RMDGCN study, with a weight parameter $\lambda$. To address limited positives and the fact that unknown pairs are not true negatives, RMDGCN applies PU learning: XGBoost iteratively scores unlabeled pairs and constructs a high-confidence adjacency matrix $\tilde{A}$ by thresholding, where a threshold of 0.6 performs best. RRS, DDS, and $\tilde{A}$ are assembled into a block-structured heterogeneous network, and an attention-based GCN performs message passing to score candidate site-disease pairs. Performance is evaluated by cross-validation using AUC and AUPR. This framework explicitly mitigates the ‘unknown is not negative’ issue and jointly models similarities and associations with a graph neural network (GNN). Its strengths include reducing the impact of false negatives and learning on a unified graph. Its limitations include sensitivity to the PU threshold and the quality of similarity measures. Hard thresholding can also add noise.

PathoRM [49] is designed for pathogenic or disease-related RNA modification site-disease association inference. It combines multi-view similarity learning with a heterogeneous graph autoencoder. It first applies a naïve multi-view learning strategy to obtain an RNA modification site similarity matrix, denoted as CSS in the original PathoRM study, and a disease similarity matrix, denoted as CDD in the original PathoRM study. The learning encourages low-rank structure and sparsity, and it seeks consensus across views. CSS and CDD are then sparsified and denoised using a threshold $\tau$. Together with the known site-disease association matrix, they are used to build an RM-disease heterogeneous graph. For inference, PathoRM uses a graph autoencoder for link prediction. The encoder can be GCN, GraphSAGE, or GIN, and it typically uses three layers to alleviate over-smoothing. It learns embeddings ${H}_S$ and ${H}_D$ for sites and diseases. A dot-product decoder reconstructs the association matrix: ${\hat{X}}^{SD}=\sigma \left({H}_S^{\top }{H}_D\right).$ To reduce noise from treating unknown pairs as negatives, PathoRM proposes a ‘guilty-by-association’ negative sampling strategy. It also uses adversarial training to improve robustness. The advantages are stable similarity estimation and end-to-end graph inference. The drawbacks are higher pipeline complexity and dependence on feature coverage, thresholds, and sampling choices.

In summary, machine learning-based methods are flexible. They integrate multi-source evidence and heterogeneous network structure, and they often achieve good ranking performance. However, results can be sensitive to similarity construction, negative sampling, and PU settings. Many studies still rely on cross-validation with sampled negatives. Therefore, careful evaluation, calibration, and independent validation are needed for practical use.

### Matrix completion-based methods

Matrix completion-based methods represent known site-disease associations as a sparse matrix $A\in{\left\{0,1\right\}}^{m\times n}$. When confidence-weighted evidence is available, $A$ can be treated as a real-valued matrix in ${\mathbb{R}}^{m\times n}$, and prediction is framed as recovering the missing entries of $A$. To incorporate prior structure, studies often construct a site similarity matrix ${W}^{SS}\in{\mathbb{R}}^{m\times m}$ and a disease similarity matrix ${W}^{DD}\in{\mathbb{R}}^{n\times n}$. These matrices are usually normalized and then sparsified to suppress noisy edges. One common approach is to retain only KNN connections, and another is to remove edges whose similarity values are below a threshold. The core assumption is that $A$ can be approximated by low-rank latent factors:


(6) \begin{equation*} A\approx U{V}^{\top },U\in{\mathbb{R}}^{m\times k},V\in{\mathbb{R}}^{n\times k} \end{equation*}

where $k$ is the latent dimension and is chosen to be much smaller than both $m$ and $n$. Similarity information is commonly incorporated via graph regularization:


(7) \begin{equation*} \underset{U,V}{\min}\parallel M\odot \left(A-U{V}^{\top}\right){\parallel}_F^2+{\lambda}_S\;\mathrm{tr}\left({U}^{\top }{L}_SU\right)+{\lambda}_D\;\mathrm{tr}\left({V}^{\top }{L}_DV\right) \end{equation*}

where $M\in{\left\{0,1\right\}}^{m\times n}$ is the observation mask that indicates which entries are observed. The mask entry ${M}_{ij}$ equals 1 for known associations and 0 for unobserved or unlabeled pairs. The symbol $\odot$ denotes the Hadamard product. The matrices ${L}_S\in{\mathbb{R}}^{m\times m}$ and ${L}_D\in{\mathbb{R}}^{n\times n}$ are graph Laplacians derived from ${W}^{SS}$ and ${W}^{DD}$, respectively. The hyperparameters ${\lambda}_S$ and ${\lambda}_D$ control the strength of smoothness regularization over the site and disease graphs, and they encourage similar sites and similar diseases to be close in the latent space. To improve robustness, some models adopt a low-rank plus sparse noise decomposition, impose norm constraints, or enforce bounded constraints on the recovered scores. Depending on the specific implementation, these matrix completion-based methods may involve matrix factorization, robust principal component analysis, or low-rank subspace recovery. Representative studies in this group include m7GDisAI, BRPCA, and SpBLRSR.

m7GDisAI [50] is designed for m7G site-disease association inference. It uses an m7G-disease dataset and constructs a heterogeneous network. The model integrates an m7G site similarity network with a disease semantic similarity network. It compares different site similarity settings, including chemical similarity CHN, CNF-based similarity CNFHN, and their combinations. It applies matrix factorization to complete unknown links in the m7G-disease association matrix constructed from the heterogeneous network. Performance is evaluated by 10-fold cross-validation and by global and local LOOCV. In an ovarian cancer case study, candidate sites are ranked. GO enrichment is performed on host genes to support biological interpretation. A key strength is its clear and simple framework. It unifies site similarity, disease similarity, and known associations and performs link completion by matrix factorization. A main limitation is that m7G-disease data are still incomplete. Performance also depends on feature design and the quality of similarity measures.

BRPCA [51] performs robust recovery on an m7G-disease heterogeneous matrix. It models the matrix as a low-rank structure plus a sparse noise term. It first constructs a site similarity network ${W}^{SS}$, a disease similarity network ${W}^{DD}$, and a binary association network ${W}^{SD}$. These parts are combined into a block heterogeneous adjacency matrix:


(8) \begin{equation*} W=\left[\begin{array}{@{}cc@{}}{W}^{SS}& {W}^{SD}\\{}{W}^{DS}& {W}^{DD}\end{array}\right],{W}^{DS}={\left({W}^{SD}\right)}^{\top } \end{equation*}

This reformulates the task. It changes the problem from recovering a highly sparse ${W}^{SD}$ to completing a denser matrix $W$. A sparsity comparison is reported. The original association matrix has about 99.41% sparsity. The heterogeneous matrix reduces sparsity to about 30.94%. The method assumes that correlated latent factors lead to a low-rank structure. It also adds a bounded constraint in $\left[0,1\right]$, which keeps predicted scores consistent with the range of similarity measures. Comparisons with RPCA and normalized RPCA show that the bounded constraint improves prediction. Evaluation includes 10-fold cross-validation and a *de novo* disease test. In the *de novo* test, all known links of one disease are removed. Candidates are ranked using only ${W}^{SS}$ and ${W}^{DD}$. This setting highlights the importance of similarity information. The main advantage is that the block matrix reduces sparsity and low-rank recovery captures shared patterns. The bounded constraint also improves interpretability. The method can rank candidates for diseases with no known links. The main limitation is sensitivity to similarity quality in *de novo* settings. The low-rank assumption may emphasize shared factors and may miss disease-specific mechanisms.

SpBLRSR [52] is a bounded low-rank subspace recovery model with a Schatten $p$-norm constraint for m7G-disease association prediction. It constructs a block matrix $H$, with site similarity and disease similarity placed in the diagonal blocks and the association matrix placed in the off-diagonal blocks. This reduces sparsity and supports settings such as leave-one-disease-out evaluation. The Schatten $p$-norm is used as a tighter approximation to rank than the nuclear norm, which can improve recovery accuracy and robustness to Gaussian noise. Evaluation reports improved performance under 10-fold cross-validation and leave-one-disease-out cross-validation. A noise contamination experiment is used to test robustness. A cancer-related case study is also included to support biological relevance. The advantage is that it combines global and local structure information. The Schatten $p$-norm and bounded constraint support accuracy, robustness, and interpretability. The limitation is higher model complexity. Results can be sensitive to hyperparameters and to the balance between global low-rank and local sparse structure. Prediction scores alone do not provide causal mechanisms. Additional biological evidence is still required.

In summary, matrix completion-based methods are simple and efficient. They naturally model missing links and can combine site similarity, disease similarity, and known associations in one framework. They often work well on sparse data, especially when block matrices or robust recovery are used. However, performance depends on the low-rank assumption and the quality of similarity matrices. Results can also be sensitive to hyperparameters and regularization weights. Predicted scores mainly reflect statistical association, so biological support and independent validation are still needed for practical use.

### Network propagation-based methods

Network propagation-based methods, including random walk with restart (RWR) and diffusion-based strategies, usually represent modification sites and diseases as a heterogeneous network. Links within the same node type are often described by a site similarity matrix ${W}^{SS}$ and a disease similarity matrix ${W}^{DD}$, whereas links between different node types are described by the known site-disease association matrix $A$. These matrices are combined into a unified graph. In some methods, a gene layer is further introduced to build a multi-layer heterogeneous network. This can improve network connectivity and provide more interpretable propagation paths. These methods typically use RWR or diffusion-based propagation to spread information on the network. Given an initial probability vector ${p}^{(0)}$, the update rule is


(9) \begin{equation*} {p}^{\left(t+1\right)}=\left(1-r\right)\hat{H}{p}^{(t)}+r{p}^{(0)} \end{equation*}

where $\hat{H}$ is the normalized transition matrix and $r$ is the restart probability. After convergence, the steady-state scores are used to rank candidate sites or site-disease pairs. In the following, we introduce DRUM and m7GDP-RW as representative examples.

DRUM [53] builds a multi-layer heterogeneous network with three node types: diseases, genes, and m6A sites. It integrates cross-layer links, such as disease-gene and gene-site relations, as well as within-layer links, such as disease-disease, gene–gene, and site-site similarity or correlation. DRUM then applies RWR on this network, using a disease node as the seed. After convergence, it ranks m6A sites by steady-state probabilities, generating a prioritized list of candidate m6A sites associated with each disease. DRUM also uses randomized networks to estimate a background distribution and to assess the statistical significance of predictions. In addition, it infers whether a candidate site is more likely to be hyper-methylated or hypo-methylated in a disease based on correlations between the site and disease-related genes. The main strengths of DRUM are that it combines multiple data sources in a single framework, integrates multi-step signals through cross-layer propagation, and directly outputs a ranked list of candidate sites. The main limitations are that performance depends on how each subnetwork is constructed and weighted. The original study used equal weights as an example and noted that these weights could be further optimized. In addition, the restart probability is set to a fixed value, which may affect robustness, especially when the network is noisy or biased.

m7GDP-RW [36] predicts associations between m7G sites and diseases. It uses known m7G-disease associations as prior information and also incorporates similarity information for m7G sites and diseases. First, it redesigns the similarity measures using known associations. It then performs clustering based on shared associations and strengthens similarity within each cluster. Next, it applies a logistic function to suppress weak similarities, which reduces noise and improves the similarity matrices. Based on the updated similarities, it builds a heterogeneous network that combines the m7G-site similarity network, the disease similarity network, and the known m7G-disease links. Finally, it runs a two-pass RWR. One pass propagates on the m7G side and the other on the disease side. The two results are then combined to produce a ranked list of candidate m7G sites for each disease. The strengths of this method are that it uses known associations to refine similarity estimation, reduces weak-similarity noise before propagation, and leverages network topology from both sides through two-pass propagation. The limitations are that the clustering and similarity updates depend on the coverage and potential bias of known associations, performance and generalization may decrease when known links are sparse or uneven, and the hyperparameters used in clustering and similarity transformation can also affect the results.

In summary, network propagation methods usually do not require end-to-end supervised training. They are simple to implement, easy to scale, and especially useful when known associations are sparse but the similarity network is reliable. Their results can be partially interpreted through propagation paths in the network. However, performance strongly depends on the quality and connectivity of the similarity network. Noisy edges or biased similarity scores can directly affect the propagation and the final ranking. In addition, these methods often output ranking scores and are generally less flexible than trainable deep models for capturing complex nonlinear interactions and integrating high-level features.

## Challenges and future directions

Although computational methods for predicting associations between RNA modification sites and diseases have made significant progress in recent years, this field still faces several challenges, including limited data availability, incomplete annotation, class imbalance, insufficient feature construction, and limited cross-platform generalization. Therefore, future research is needed to improve multimodal information integration, develop unified modeling frameworks, explore weakly supervised learning strategies, and enhance model interpretability. At the same time, it is necessary to reduce the sensitivity of existing methods to feature coverage, threshold settings, and sampling strategies to improve the robustness and reproducibility of prediction results. The main challenges and possible directions are discussed below:

(1) Regarding RNA modification site data, it is necessary to further expand the scale and diversity of available datasets. On the one hand, with the continued development of high-throughput sequencing and modification detection technologies, more abundant and higher-quality modification site data are expected to become available. This progress will help alleviate issues such as limited sample size, incomplete annotation, and class imbalance. On the other hand, most existing studies focus on a small number of RNA modification types with relatively sufficient data. For disease association prediction tasks, integrating different RNA modification types and extending current research to a broader range of modifications may improve the generality and applicability of RNA modification site-disease association prediction methods. In addition, integrating modification sites with regulatory factors, genetic variation information, and disease phenotypes may help reveal their potential functional roles and disease relevance within a more comprehensive biological regulatory network.
(2) Regarding feature construction and information integration, future studies can move beyond representations that rely mainly on local sequence information and incorporate more context-aware multimodal features. In addition to sequence features, functional region annotations, regulatory factor binding evidence, variation-induced perturbation signals, and tissue- or cell-type-specific information can provide important biological context for RNA modification sites. In particular, RNA secondary structure and structural accessibility are closely related to modification occurrence and functional regulation. Integrating RNA structural features with sequence and regulatory context information into predictive models may better reflect the biological environment of modification sites and further improve prediction accuracy and generalization performance.
(3) Regarding model performance and learning paradigms, future research should place greater emphasis on robust modeling under sparse and noisy data conditions while continuously improving model architectures and computational strategies. Some existing studies have attempted to combine deep learning methods with heterogeneous network modeling frameworks to jointly capture sequence features and multi-source association information, and these studies suggest that the two approaches are complementary for representation learning and global relationship modeling. However, current fusion strategies still leave room for improvement in terms of model consistency, training stability, and robustness to noisy and sparse associations. Deep learning continues to advance in sequence representation learning and nonlinear feature extraction, while heterogeneous network-based methods remain valuable for integrating multi-source relationships, exploiting global topological information, and alleviating data sparsity. Future studies may further integrate sequence features, network topology, and hierarchical biological evidence within weakly supervised learning frameworks to identify more stable and biologically meaningful association patterns.

## Conclusion

RNA post-transcriptional modifications, such as m6A, m1A, and m7G, are dynamic, reversible, and context dependent. They regulate gene expression homeostasis by affecting RNA splicing, export, stability, and translation. Dysregulation of these modifications is widely linked to the onset and progression of many diseases. Therefore, predicting associations between RNA modification sites and diseases can help clarify regulatory networks and disease mechanisms. It may also support the identification of potential biomarkers, therapeutic targets, and drug candidates. In recent years, advances in high-throughput sequencing and epitranscriptomics have generated more data on modification sites and their disease relevance. These data provide a foundation for developing computational prediction methods. In this review, we summarize key resources and common technical routes for predicting RNA modification site-disease associations. We describe databases and data sources, strategies to construct site and disease similarity, approaches to build heterogeneous or multi-layer networks, and widely used prediction frameworks and application settings. We also summarize the main ideas, suitable conditions, and limitations of existing methods.

Most current methods follow a general workflow that includes data integration, similarity construction, network modeling, and model inference. These methods have produced useful results, but several challenges remain. Known associations are limited and highly imbalanced, and negative samples are often undefined. As a result, performance can be sensitive to sampling and evaluation choices. Multi-source data include tissue- and cell-type differences and noise, which can reduce generalization across datasets and application scenarios. Studies often use different data splits, hyperparameter settings, and evaluation metrics, which limits reproducibility and makes fair comparisons difficult. In addition, model interpretability and experimental validation are still limited, which constrains biological insight and clinical translation. Future work can focus on three aspects: data, association networks, and validation. Collecting more confirmed site-disease associations will enable more complete similarity information for sites and diseases. More comprehensive heterogeneous networks may help identify additional disease-related candidate modification sites. Integrating multi-omics data under different biological conditions may further reveal tissue-specific and stage-specific modification patterns. Finally, experimental validation is essential to increase confidence in computational predictions.

**Key points**

- This review summarizes the main data resources for RNA modification site-disease association prediction and classifies existing computational methods into three categories: machine learning-based methods, matrix completion-based methods, and network propagation-based methods.
- We highlight the main ideas, strengths, limitations, and application settings of representative methods in this field.
- We also discuss current challenges and future directions, including limited known associations, class imbalance, ambiguous negative samples, multimodal integration, and experimental validation.

## Data Availability

Details about the data discussed in this study have been incorporated into the article. No additional data were generated for this study.
